# Shared Transcriptional Machinery at Homologous Alleles Leads to Reduced Transcription in Early *Drosophila* Embryos

**DOI:** 10.3389/fcell.2022.912838

**Published:** 2022-07-11

**Authors:** Hao Deng, Bomyi Lim

**Affiliations:** Department of Chemical and Biomolecular Engineering, University of Pennsylvania, Philadelphia, PA, United States

**Keywords:** *Drosophila* embryos, MS2, PP7, transcription, enhancers, live imaging

## Abstract

The mechanism by which transcriptional machinery is recruited to enhancers and promoters to regulate gene expression is one of the most challenging and extensively studied questions in modern biology. We explored the possibility that interallelic interactions between two homologous alleles might affect gene regulation. Using an MS2- and PP7-based, allele-specific live imaging assay, we visualized *de novo* transcripts of a reporter gene in hemizygous and homozygous *Drosophila* embryos. Surprisingly, each homozygous allele produced fewer RNAs than the corresponding hemizygous allele, suggesting the possibility of allelic competition in homozygotes. However, the competition was not observed when the enhancer-promoter interaction was weakened by placing the reporter construct in a different chromosome location or by moving the enhancer further away from the promoter. Moreover, the reporter gene showed reduced transcriptional activity when a partial transcription unit (either an enhancer or reporter gene only) was in the homologous position. We propose that the transcriptional machinery that binds both the enhancer and promoter regions, such as RNA Pol II or preinitiation complexes, may be responsible for the allelic competition. We showed that the degree of allelic interference increased over developmental time as more Pol II was needed to activate zygotic genes. Such allelic competition was observed for an endogenous gene as well. Our study provides new insights into the role of 3D interallelic interactions in gene regulation.

## Introduction

Enhancers, which contain multiple binding sites for sequence-specific transcription activators and repressors, determine when and where a target gene should be transcribed (Levine and Tjian, 2003; Levine et al., 2014). Missense mutations in enhancers or disruptions in enhancer-promoter interactions often result in ectopic or lost expression of target genes (Halder et al., 1995; Riedel-Kruse et al., 2007). Many of these genetic perturbations in enhancers are associated with various disease phenotypes, emphasizing the role of precise enhancer-promoter communications in normal development (Hnisz et al., 2013; Miguel-Escalada et al., 2015). Extensive studies have been conducted to elucidate the mechanism of enhancer-mediated transcriptional regulation, and yet, there remain more questions to be explored. For example, how do multiple enhancers drive target gene expression in the same tissue while coordinating among themselves to ensure access to the target promoter (Perry et al., 2011; Lim et al., 2018a; Berrocal et al., 2020)? A live imaging study on early *Drosophila* embryos demonstrated that some enhancers work additively with each other, while others work synergistically or competitively, such that multiple enhancers can drive higher or lower transcriptional activity than a single enhancer (Bothma et al., 2015). Additionally, a single enhancer can interact with multiple promoters, sometimes activating the target promoter on the homologous allele in *trans* (Fukaya et al., 2016; Lim et al., 2018b; Levo et al., 2022). These results indicate that enhancer-promoter communication involves interactions among multiple transcriptional regulatory units, a far more dynamic process than previously envisioned.

In parallel, multiple studies have shown that transcriptional regulators like RNA polymerase II (Pol II), Mediators, pre-initiation complexes (PICs), and transcription factors (TFs) form clusters at active transcription loci (Kato et al., 2012; Cisse et al., 2013; Cho et al., 2016; Wollman et al., 2017; Chong et al., 2018; Sabari et al., 2018). It has been suggested that TFs cluster at enhancers and Pol II/Mediator cluster at promoters, forming active hubs to regulate transcription (Tsai et al., 2017; Boija et al., 2018). Indeed, studies in *Drosophila* embryos showed that highly concentrated local clusters of the pioneer factor Zelda (Zld) at transcription loci facilitate the binding of Bicoid (Bcd) and Dorsal (Dl) activators to the target DNA (Mir et al., 2017, 2018; Yamada et al., 2019). This idea of a “transcription hub” can also explain previous findings on multivariate enhancer-promoter interactions where one enhancer can co-activate two target promoters in *cis* as well as in *trans* (Fukaya et al., 2016; Lim et al., 2018b; Levo et al., 2022). Altogether, these studies propose that the clustering of transcriptional machinery in a nucleus plays an important role in enhancer-mediated gene regulation.

In this study, we provide evidence that two homologous alleles may compete and affect the level of RNA production. Using allele-specific MS2- and PP7- based live imaging methods in early *Drosophila* embryos, we measured the transcriptional activity of a reporter gene in one allele from homozygous and hemizygous embryos. Surprisingly, we found that one homozygous allele produced fewer RNAs than its hemizygous counterpart. This decrease was manifested mainly as a change in transcriptional amplitude, implying that the number of RNA Pol II loaded to each allele was reduced in homozygotes. Interestingly, this allelic competition at the homologous locus was not observed in the absence of strong enhancer-promoter interactions.

To examine which transcriptional machinery might affect interallelic interactions, we measured the transcriptional activity of a reporter gene when the homologous allele contains only an enhancer or a promoter-reporter gene. Unexpectedly, both the “Enhancer Only” and the “Promoter Only” allele on the homologous position were sufficient to decrease the transcriptional activity. This implies that the transcriptional machinery binding to both the enhancer and promoter plays a role in the allelic competition. Based on these results, we propose that homologous alleles may share the same local transcription hub and that each allele produces a reduced number of RNAs when the number of Pol II in the hub is limiting—especially upon strong enhancer-promoter interactions. Indeed, we showed that the competition was observed only in the nuclear cycle 14 (NC14) when massive zygotic genome activation occurs. Lastly, we demonstrated that endogenous *snail* alleles also interfere with each other. Our study provides new insights into a mechanism of transcriptional regulation in 3D environments.

## Materials and Methods

Detailed Materials and Methods are in the Supplementary Material.

## Results

### MS2- and PP7-Based Labeling of two Homologous Alleles

**FIGURE 1:**
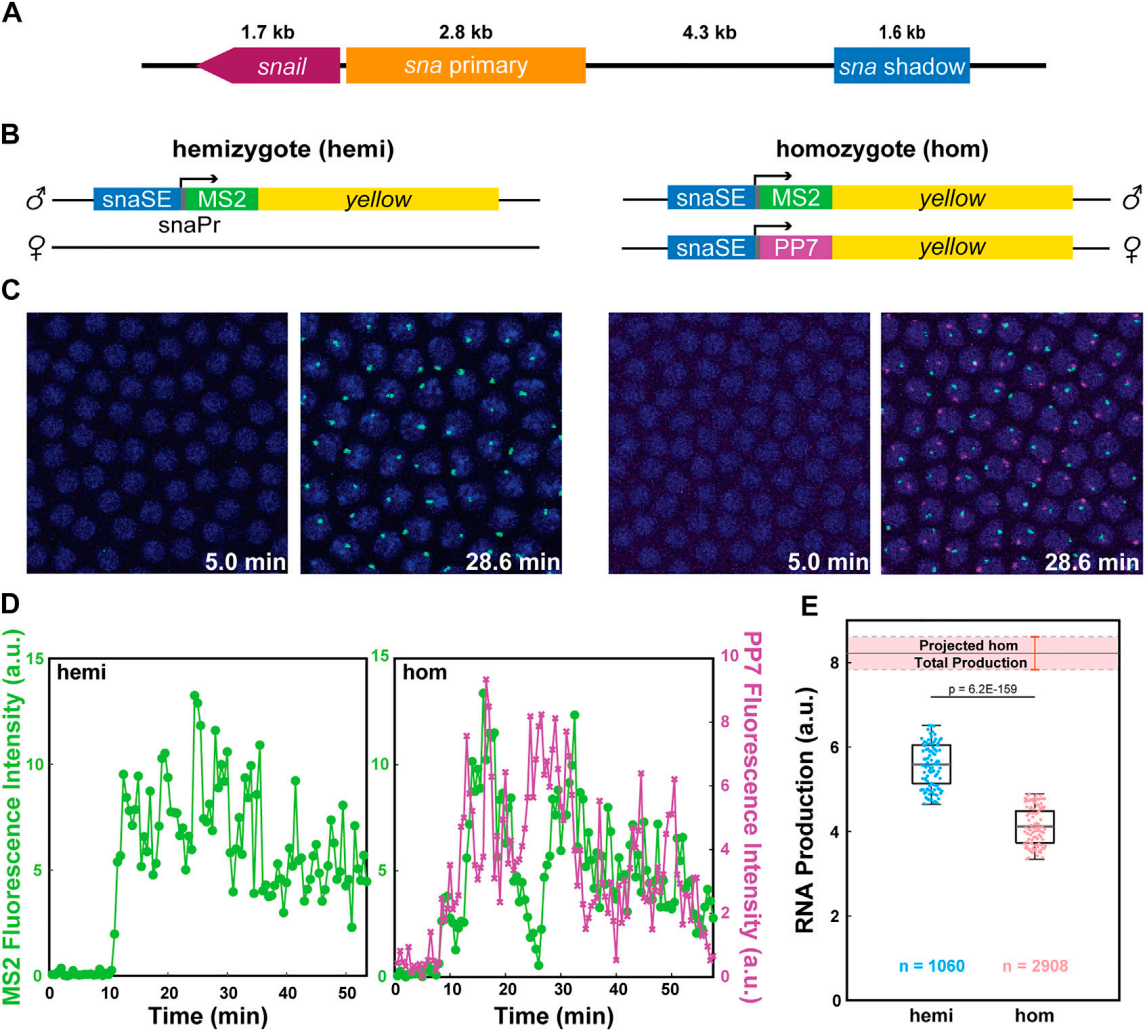
Allelic competition between the homologous alleles reduces transcriptional activity. **(A)** Schematic of the *snail* gene (*sna*), the primary enhancer (snaPE), the shadow enhancer (snaSE). **(B)** Schematic of the hemizygous and homozygous snaSE>*yellow* constructs. snaSE is placed right upstream of the *sna* promoter. 24 copies of MS2 and PP7 sequences were inserted for allele-specific visualization of the transcriptional activity. The paternal allele contains the *MS2-yellow* reporter gene for both hemizygotes and homozygotes. **(C)** Representative snapshots of hemizygous and homozygous embryos containing the snaSE>*yellow* transgene. The time indicates minutes after the onset of NC14. Fluorescence puncta (green—*MS2-yellow;* red—*PP7-yellow*) indicate active nascent transcripts. Nuclei were visualized with His2Av-eBFP2 (blue). **(D)** Representative transcriptional trajectories from a nucleus of hemizygous and homozygous embryos shown in (C). Transcriptional activity is proportional to the fluorescence intensity. The total RNA production of each allele can be estimated by measuring the area under the transcription trajectory. **(E)** Boxplot showing RNA production of the snaSE>*MS2-yellow* allele in hemizygous and homozygous embryos. The scatter points show RNA production of two hundred random nuclei from the analysis. The boxplots show that the *MS2-yellow* allele from homozygotes produced approximately 25% fewer RNAs than the one from hemizygotes. The band above the homozygote boxplot shows the projected total RNA production of a homozygous embryo, which is obtained by doubling the RNA production of one allele in homozygotes. Hemizygotes produce around 70% of the total RNA production of homozygotes. n indicates the number of analyzed nuclei from 4 and 10 biologically replicate embryos of each genotype respectively. The box indicates the 25%, 50% and 75% quantile, and the whiskers extend to the 10th and 90th percentile of each distribution.

We compared the transcriptional activity of reporter genes driven by the well-characterized *snail* shadow enhancer (snaSE) between hemizygous and homozygous embryos to test the possibility that homologous alleles may interact with each other (Figure 1A) (Perry et al., 2010). MS2- and PP7-based live imaging methods, which were successfully implemented in *Drosophila* embryos and other tissues, were used to visualize nascent transcripts (Bertrand et al., 1998; Larson et al., 2011; Coulon et al., 2013; Fukaya et al., 2017; Chen et al., 2018). We generated transgenic lines where the snaSE and the 100-bp core promoter of *sna* drive expression of the *MS2-yellow* and the *PP7-yellow* reporter gene. Upon transcription, the MS2 or PP7 sequence forms a stem-loop structure, which can be recognized by two copies of the MS2 coat protein (MCP) or the PP7 coat protein (PCP), fused with GFP or mCherry, respectively (Figure 1B). The binding of MCP-GFP or PCP-mCherry to the transcribed MS2 or PP7 stem loops allows visualization of *de novo* transcripts in living embryos (Figure 1C) (Lim et al., 2018b). The reporter genes were inserted into a specific location in the 3rd chromosome using PhiC31-mediated site-directed transgenesis (Groth, 2004; Bischof et al., 2007).

To distinguish transcriptional activities from each allele in homozygous embryos, we crossed nos>MCP-GFP, PCP-mCherry/snaSE*>PP7-yellow* females with snaSE*>MS2-yellow* homozygous males. Fifty percent of the progeny have two copies of the *yellow* reporter gene, each marked with *PP7* and *MS2* stem-loops (homozygotes). The other 50% have one copy of the *yellow* reporter gene marked with MS2 stem-loops (hemizygotes) (Figure 1C and Supplementary Movie S1). To note, the paternal allele carries the *MS2-yellow* for both homozygous and hemizygous embryos.

### Live Imaging Reveals a Possibility of Allelic Competition Between the Homologous Alleles

Since our live imaging methods provide instantaneous transcriptional activity as a function of time, we can estimate total RNA production by measuring the area under the transcriptional trajectory of each nucleus (Figure 1D and Supplementary Figure S1). Theoretically, if the homologous alleles behave independently of each other, one single allele from homozygous embryos should produce a comparable number of RNAs as the hemizygous allele. In the case of interallelic interaction, the transcriptional activity of each homozygous allele would differ from that of the hemizygous allele. To our surprise, the *MS2-yellow* allele in homozygous embryos produced about 25% fewer RNAs than the hemizygous *MS2-yellow* allele (Figure 1E).

Considering we obtained the fluorescent signals solely from the paternal allele of the homozygotes, we acknowledge that there could be a bias in RNA production between the maternal and the paternal alleles where the alleles may complement each other instead of interfering with each other. To address this potential bias, we generated two homozygous constructs, one with the maternal *MS2-yellow* and the other with the paternal *MS2-yellow*. We confirmed that the *MS2-yellow* transcriptional activity does not change between maternal and paternal alleles of homozygous embryos, suggesting that the total RNA production can be estimated by doubling the RNA production of one allele (Supplementary Figure S2). The estimated total RNA production of homozygotes was about 1.5 times the RNA production in hemizygotes, rather than twice, which is expected if there were no interallelic interaction (Figure 1E). In sum, our allele-specific live imaging assays suggest that the homologous alleles of snaSE>*yellow* reporter genes may interact in *trans* and inhibit each other, resulting in lower RNA production than expected.

**FIGURE 2:**
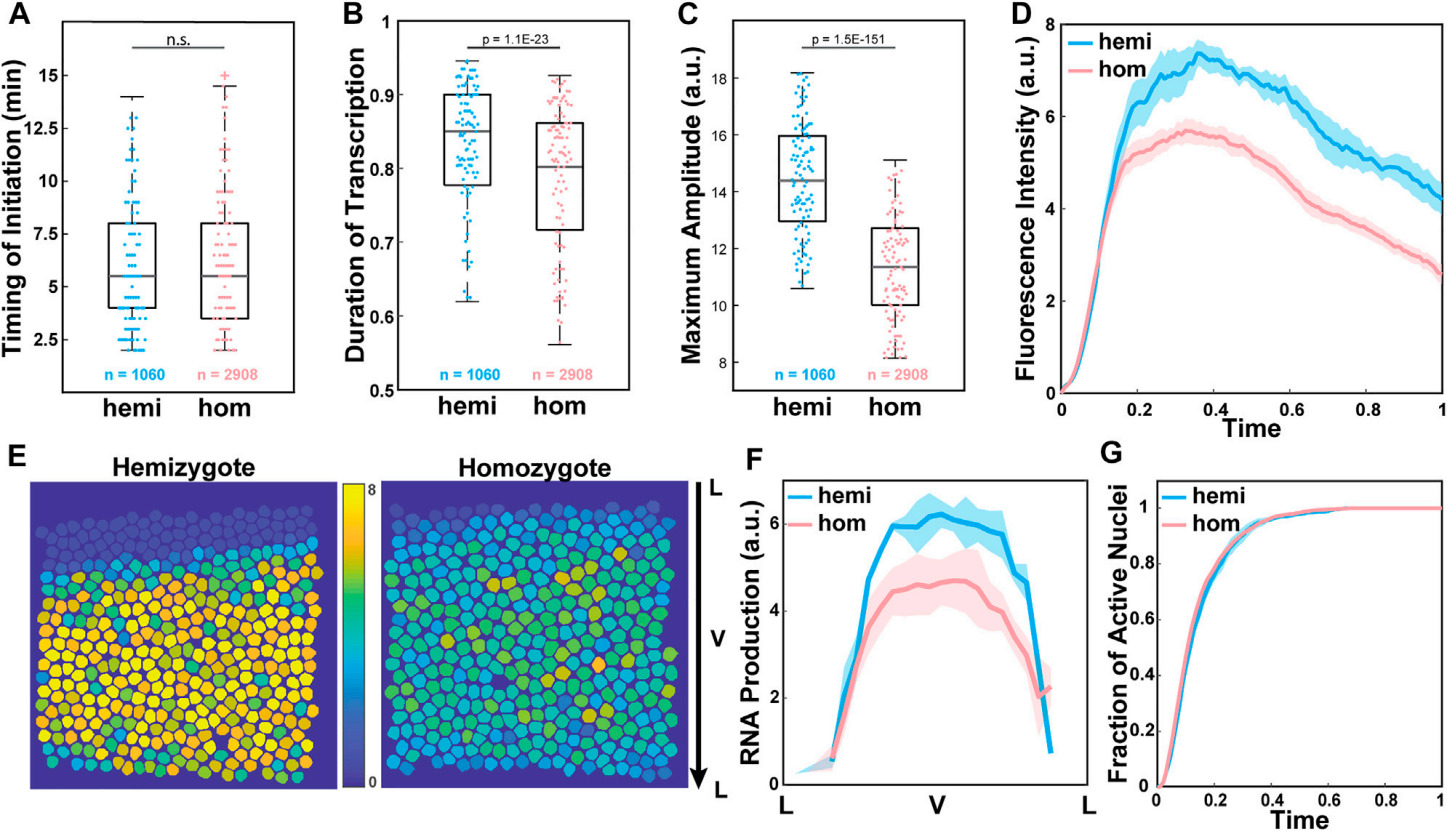
Low transcriptional amplitude caused reduced RNA output in the homozygous alleles. **(A)** Boxplot of the timing of transcription initiation for hemizygotes (hemi) and homozygotes (hom) expressing snaSE>*yellow*. For both genotypes, transcription was initiated about 6 min after the onset of NC14. n indicates the number of analyzed nuclei from 4 and 10 biologically replicate embryos of each genotype, respectively. **(B)** Boxplot of the duration of active snaSE>*MS2-yellow* transcription in NC14, where total duration is scaled to one. The hemizygous and the homozygous alleles spend comparable time in the active transcription state. **(C)** Boxplot of the average amplitude of *MS2-yellow* fluorescent intensity in snaSE>*yellow* hemizygotes and homozygotes. The amplitude in homozygous embryos is about 20% lower than the one in hemizygous embryos. **(D)** Average transcriptional trajectories of active nuclei from hemizygous (blue) and homozygous (red) embryos. The main difference between the genotypes is the average amplitude. **(E)** Heat maps of a representative snaSE>*yellow* hemizygous (left) and homozygous (right) embryo showing the accumulated RNA production of the *MS2-yellow* allele in NC14 of all nuclei within the *sna* expression domain. The RNA production is reduced throughout the ventral side of the homozygous embryo. The snapshot shows a ventral view of an embryo. **(F)** Average RNA production of one allele in hemizygotes (blue) and homozygotes (red) expressing the snaSE>*MS2-yellow* reporter gene along the dorsoventral axis of an embryo. The RNA production is reduced throughout the domain in homozygotes yet the *sna* expression boundary is not narrowed. **(G)** Plot of the cumulative fraction of active nuclei over the duration of NC14 in hemizygotes (blue) and homozygotes (red). Both genotypes produce RNAs with similar kinetics of transcriptional activation. The number of analyzed nuclei is the same as the one shown in Figure 1E. For boxplots in (A–C), the scatter points indicate values from 200 randomly selected nuclei used in the analysis. The box indicates the 25%, 50% and 75% quantile, and the whiskers extend to the 10th and the 90th percentile of each distribution. The error bar in (D), (F) and (G) represents the Standard Error of Mean (SEM) for 4 and 10 biologically replicate embryos for hemizygous and homozygous snaSE>*yellow* embryos, respectively.

We investigated the source of observed reduced RNA production by analyzing three parameters extracted from our single-cell resolution live imaging data. The lower RNA production can be caused by different factors during transcription. First, the delayed onset of transcription due to a lag in enhancer-promoter interactions could cause lower transcriptional activity. Alternatively, infrequent Pol II loading to the promoter could result in less frequent transcriptional bursting, resulting in a shorter duration of active transcription and reduced RNA production. Lastly, a reduction in the number of Pol II loaded to the promoter could lead to a decrease in transcriptional amplitude and a reduction in RNA production. To distinguish these factors, we measured three parameters: (i) the timing of transcription initiation, (ii) the duration of active transcription, and (iii) the average amplitude of transcription in each transcriptionally active nucleus. We found that transcription was initiated about 6 min after the onset of NC14 in both hemizygous and homozygous embryos (Figure 2A). The duration of active transcription was also comparable between the two genotypes, with about a 5% shorter duration for the homozygous allele (Figure 2B). Unlike these two parameters that showed a minimal effect, the average amplitude of transcriptional activity was significantly lower in the homozygous *MS2-yellow* allele than in the hemizygous allele (Figure 2C). Indeed, when we examined the average trajectory of the homozygous and the hemizygous *MS2-yellow* allele, the hemizygous allele maintained a higher amplitude till the end of NC14 (Figure 2D).

In addition to the single-cell analysis, we analyzed if all the nuclei within the *sna* expression domain uniformly exhibited lower transcriptional amplitude, or if the boundary nuclei where the concentration of the Dorsal activator is lower showed a greater reduction in amplitude. Similar to what we observed in the single-cell analysis, the level of RNA production decreased, but the overall width and pattern of the *sna* boundary remained unchanged (Figures 2E,F). We also measured the cumulative fraction of active nuclei over time, and both the homozygous and hemizygous *MS2-yellow* allele exhibited similar kinetics of transcriptional activation (Figure 2G). This result indicates that the rate of forming the *sna* expression boundary is similar between the two genotypes. Taken together, our analysis suggests that the alleles may compete in *trans* throughout the *sna* expression domain, mainly by modulating the transcriptional amplitude.

### Weaker Enhancer-Promoter Interactions do not Result in Allelic Interference but Alleles With Partial Transcription Units Still Compete With Each Other

**FIGURE 3:**
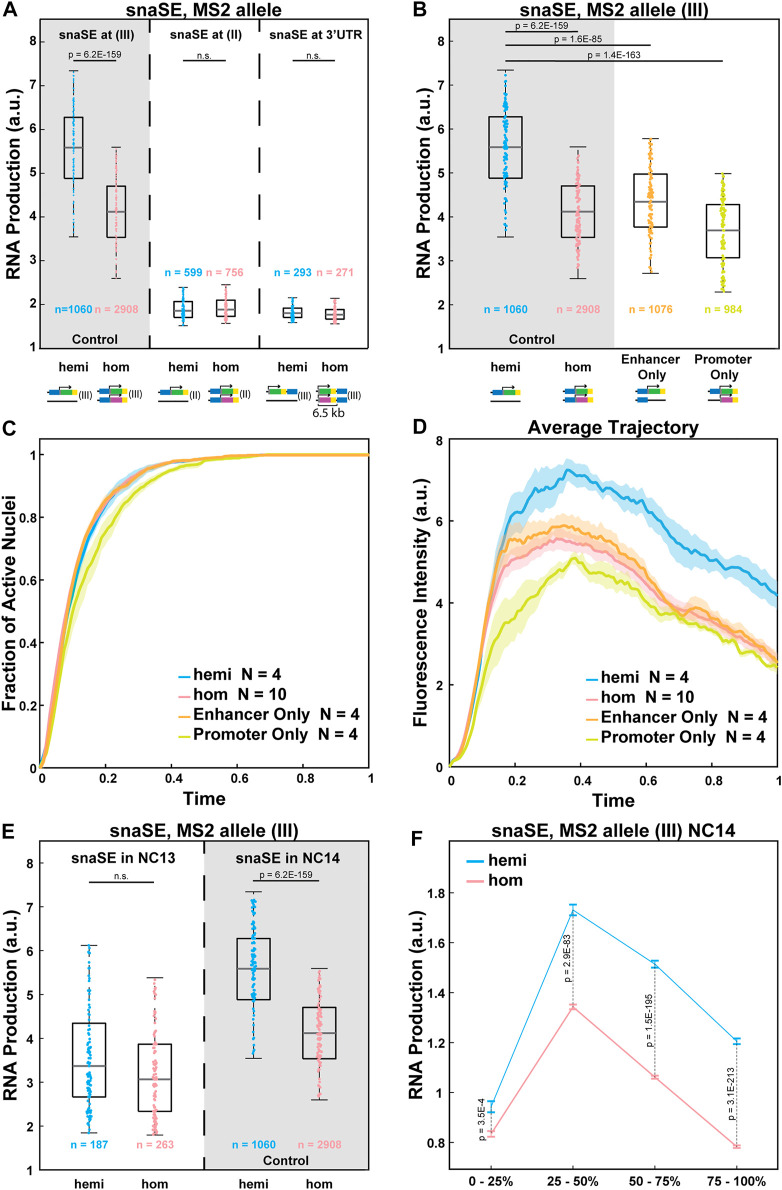
Allelic competition is not observed when the enhancer-promoter interactions are weakened. **(A)** Plots comparing the RNA production of the *MS2-yellow* allele between the homozygous and the hemizygous constructs of snaSE>*yellow* (III), snaSE>*yellow* (II) and snaSE3’>*yellow*. The homozygous alleles of the latter two constructs do not compete with each other. The simplified schematics of the constructs are shown under each genotype. n indicates the number of analyzed nuclei from 4, 10, 4, and 4 biologically replicate embryos of each genotype, respectively. **(B)** Boxplots showing RNA production of the *MS2-yellow* allele from the hemizygous, homozygous, Enhancer Only and Promoter Only embryos containing snaSE>*yellow*. The simplified schematics of the constructs are shown under each genotype. The *MS2-yellow* allele exhibits significant reduction in transcriptional activity in the presence of an Enhancer Only or the *PP7-yellow* reporter gene only on the homologous position. n indicates the number of analyzed nuclei from 4, 10, 4 and 4 biologically replicate embryos of each genotype, respectively. **(C)** Plot of the cumulative fraction of active nuclei over the duration of NC14 in hemizygotes (blue), homozygotes (red), Enhancer Only (orange) and Promoter Only (yellow). N indicates the number of biologically replicate embryos of each genotype, respectively. **(D)** Plot of the average transcriptional trajectories of active nuclei in hemizygotes (blue), homozygotes (red), Enhancer Only (orange) and Promoter Only (yellow). N indicates the number of biologically replicate embryos of each genotype, respectively. **(E)** Boxplot showing the RNA production of the snaSE>*MS2-yellow* during NC13 compared to the production during NC14. No allelic competition is observed in NC13. n indicates the number of analyzed nuclei from 3, 5, 4 and 10 biologically replicate embryos of each genotype, respectively. For all boxplots, the box indicates the 25%, 50% and 75% quantile, and the whiskers extend to the 10th and 90th percentile of each distribution. The scattered points indicate values from 200 (B, D) or 100 (E) randomly selected nuclei used in the analysis. **(F)** Plot showing the RNA production of the snaSE>*MS2-yellow* during NC14. RNA production was measured in four temporal classes in NC14 by dividing the duration of NC14 into four. In early NC14, transcriptional activities of both genotypes are comparable to each other. Later in NC14, however, a reduced expression is observed in homozygotes with a greater difference towards the end of NC14. The data points show the mean and the error bars show the standard errors of the dataset.

We next examined the potential mechanisms of the observed allelic competition. One possible explanation is that transcription factors that are available to bind to enhancers are limiting. A recent study showed that a limiting number of transcription factors could lead to reduced RNA production from the homozygous allele (Waymack et al., 2021). To further test this idea, we varied the strength of enhancer-promoter interactions without changing the enhancer sequence. Since the same transgene could have different degrees of enhancer-promoter interactions and produce different amounts of RNA depending on the chromosomal location (Lewis, 1954; Wallrath and Elgin, 1995), we inserted the strong snaSE>*yellow* constructs to the 2nd chromosome using the VK00002 line instead of the 3rd chromosome position used as the control (Figure 3A) (Venken et al., 2009). The transgene was inserted into a homologous position in the 2nd chromosome. In this chromatin context, the transcriptional activity was reduced by about 60% compared to the control (Figure 3A).

Transcriptional activity of the reporter gene can also be reduced by increasing the enhancer-promoter distance, thereby weakening their interactions. Therefore, we created a construct where snaSE was inserted at the 3′UTR of the reporter gene, around 6.5 kb downstream of the promoter (Figure 3A). While the enhancer-promoter distance is often correlated with the degree of transcriptional activity (Oudelaar et al., 2019; Zuin et al., 2022), it is not always the case depending on the insulator localization and the 3D genome folding context (Symmons et al., 2016). In our case, however, the enhancer and its target promoter (6.5 kb away) are still within the same TADs with no insulator in between. Hence, the enhancer can directly interact with the target promoter with a lower frequency than the control construct where the enhancer and the promoter are adjacent to one another. The average transcriptional activity was reduced by 64% compared to the snaSE>*MS2-yellow* control (Figure 3A).

In both constructs where we reduced the enhancer-promoter interaction and hence the transcriptional activity, there was no sign of interallelic competition. The homozygous and hemizygous *MS2-yellow* allele produced a comparable number of RNAs (Figure 3A). Since we used the same *snaSE* sequence in the control and the weakened-interaction constructs, a similar number of transcription factors would have bound to the enhancer in these constructs. We acknowledge that there is a possibility that the number of transcription factors bound to the enhancer is different due to a different chromatin landscape in the 2nd chromosome construct. Yet, no allelic competition in the construct with a greater enhancer-promoter distance in the same chromosomal position as the control indicates that the number of transcription factors is not the only limiting factor that causes the allelic interference.

Another possibility is that the limiting number of Pol II and other PIC molecules induced the reduction in transcriptional activity in homozygous embryos. With recent studies on enhancer RNA, it is known that Pol II binds to both the enhancer and the promoter regions to initiate transcription at both locations (Kim et al., 2010; Adelman and Lis, 2012; Savic et al., 2015). It was also shown that in early *Drosophila* embryos, the amount of TATA-Binding Protein (TBP) and TAFII is limiting (Zhou et al., 1998; Mannervik, 1999). Based on such previous studies, we hypothesized that two homologous alleles may share the same transcription hub where the number of Pol II and PIC factors are limiting, resulting in reduced transcriptional activity (see Discussion).

Two additional constructs were designed to test this idea. In these constructs, one allele remains the same with an intact *sna* shadow enhancer, 100-bp core *sna* promoter, and the *MS2-yellow* reporter gene, while the homologous allele contains either only the *sna* shadow enhancer without the reporter gene (“Enhancer Only”) or the promoter-reporter gene cassette without the *cis-*linked enhancer (“Promoter Only”) (Figure 3B). If the number of transcription factors is limiting, interference will occur for the “Enhancer Only” construct since transcription factors will still bind to the enhancer on both alleles. On the other hand, the interference should not be observed for the “Promoter Only” construct, as transcription factors do not bind to the core promoter, and the *MS2-yellow* allele is expected to behave similarly to the hemizygous allele.

Surprisingly, we found that the transcriptional activity of the *MS2-yellow* allele from both the “Enhancer Only” and the “Promoter Only” constructs behaved like the *MS2-yellow* allele from homozygous constructs, exhibiting reduced transcriptional activity compared to the hemizygotes (Figure 3B). We also examined the kinetics and the average trajectory of “Enhancer Only” and “Promoter Only” constructs and compared them to the hemizygotes and homozygotes of snaSE*>MS2-yellow* in the 3rd chromosome (control). All four constructs exhibited similar kinetics of transcriptional activation with a slight delay in activation in the “Promoter Only” construct (Figure 3C). The average transcriptional trajectories of these partial constructs were also comparable to the homozygotes, with slightly lower amplitude in the “Promoter Only” (Figure 3D). Our results show that the alleles can interfere with each other even if the homologous allele has only a partial transcription unit. This suggests that molecules that bind to both enhancers and promoters, such as Pol II and PIC factors, may play a role in allele competition.

### Allelic Interference is Observed Only in Late NC When More Pol II is Needed

We wanted to further test the idea that the limiting number of local Pol II and PIC hubs prompts allelic interference. Since the demand for Pol II increases over NCs, we compared the transcriptional activity between hemizygous and homozygous embryos in early and late NCs. In NC13, around 950 zygotic genes are activated, while around 3,500 genes are activated in NC14 (Kwasnieski et al., 2019). We hypothesized that such massive activation of the zygotic genome in NC14 could greatly consume local Pol II and PICs in each hub, leading to reduced transcriptional activity from each allele. Indeed, we found that during NC13, the transcriptional activity of the *sna*SE>*MS2-yellow* allele was comparable between hemizygous and homozygous embryos (Figure 3E). We then evenly divided NC14 into four temporal classes (0–25%, 25–50%, 50–75%, and 75–100% of NC14) and examined how the RNA production differs between the hemizygotes and homozygotes throughout NC14. RNA production was similar between the two genotypes in early NC14. However, the homozygous *MS2-yellow* allele produced fewer RNAs compared to the hemizygous *MS2-yellow* allele, showing larger differences in late NC14 (Figure 3F). Therefore, our observations of allelic competition in NC14, but not in NC13, support the hypothesis that the limiting number of local Pol II and PICs could lead to the observed allelic competition.

### Endogenous *sna* Also Exhibits Allelic Competition

**FIGURE 4:**
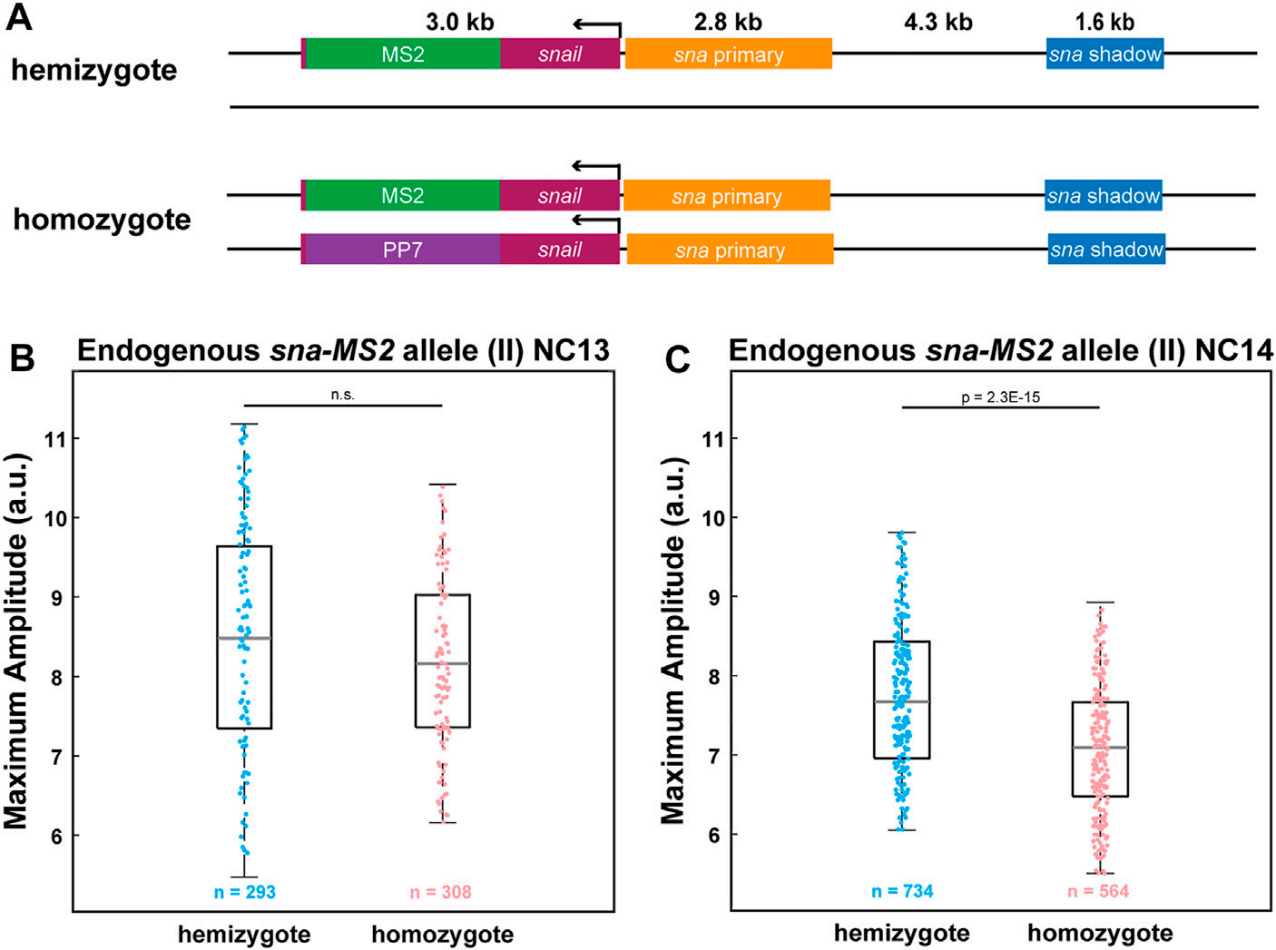
Allelic competition is observed for endogenous *sna*. **(A)** Schematic of the hemizygous and homozygous endogenous *sna* constructs. MS2 or PP7 stem loops are inserted into the 3′UTR of the endogenous *sna* via CRISPR-mediated genome editing. **(B–C)** Boxplot of the transcriptional amplitude of the hemizygous and homozygous *sna-MS2* alleles during (B) NC13 and (C) NC14. No allelic competition is observed in NC13. However, amplitude of the homozygous *sna-MS2* allele is about 10% lower than the hemizygous allele’s amplitude in NC14. n indicates the number of analyzed nuclei from 2, 2, 3, and 3 biologically replicate embryos of each genotype, respectively . For boxplots in (B-C), the scatter points indicate values from 100 (B) or 200 (C) randomly selected nuclei used in the analysis. The box indicates the 25%, 50% and 75% quantile, and the whiskers extend to the 10th and the 90th percentile of each distribution.

So far, we have relied on transgenic reporter genes to provide evidence that the two homologous alleles compete with each other. We wondered if a homozygous allele of an endogenous gene also produces fewer RNAs than a heterozygous allele. Using CRISPR/Cas9-mediated genome editing, we inserted MS2 and PP7 stem loops to the 3′ UTR of the endogenous *sna* to generate *sna-MS2* and *sna-PP7* lines (Figure 4A). By crossing *sna-MS2* homozygous flies with *sna-PP7/CyO* flies, we obtained either hemizygous *sna-MS2/CyO* or homozygous *sna-MS2/sna-PP7* embryos. Similar to the transgenic lines, we found that the endogenous *sna* alleles also compete with each other such that the homozygous *sna-MS2* allele produces fewer RNAs than the hemizygous allele (Figure 4C). Moreover, this interference was only observed in NC14 but not in NC13, agreeing with the results from the transgenic lines (Figures 3E, 4B). Our results with endogenous *sna* suggest that the allelic competition may be a general feature of transcriptional regulation for some strongly expressed genes. Taken together, we believe that the localized cluster of Pol II and PICs along with specific transcription factors form “transcription hubs” within a nucleus, capping the total RNA production level for some strong genes and resulting in reduced transcriptional activity of homozygous alleles.

## Discussion

Here, we have shown that homozygous alleles may interfere with each other to produce fewer RNAs per allele than a hemizygous allele. Strikingly, this decrease in RNA production was observed even when the homologous allele contained only a partial transcription unit such as an enhancer or reporter gene only. We have presented evidence to support the hypothesis that the local concentration of Pol II in transcription hubs may be limiting, leading to allelic competition and reduced transcriptional activity.

A recent study demonstrated a similar reduction of transcriptional activity in homozygotes compared to the hemizygotes, using reporter genes driven by the *Krüppel* enhancers. Inserting an array of Bcd or Zld TF binding sites on the homologous position was sufficient to reduce the transcriptional activity of the reporter gene, suggesting that the limiting number of TFs may induce allelic competition (Waymack et al., 2021). This idea is in agreement with our finding that the stronger *sna* shadow enhancer exhibits allelic interference. However, we also showed that the same *sna* shadow enhancer does not drive allelic interference when the enhancer-promoter interaction was weakened by moving the transgene to a different chromosome or by increasing the enhancer-promoter distance (Figure 3B). Moreover, transcriptional activity from the snaSE>*MS2-yellow* was reduced when the homologous allele had only the core promoter and the *MS2-yellow* reporter gene without the enhancer (Figure 3C). Since the site-specific TFs like Dl do not bind to the 100bp-core promoter region, we do not think that the number of site-specific TFs is the only limiting factor responsible for the allelic interference.

Instead, we suggest that RNA Pol II levels may be also limiting for strongly activated genes. Previous studies showed that the level of TATA-Binding Protein (TBP) and TAFII is limiting in *Drosophila* (Colgan and Manley, 1992; Aoyagi and Wassarman, 2001). For example, one study in early *Drosophila* embryos showed that in the sensitized Dl heterozygous background, TBP or TAFII deletion in one allele leads to defects in *sna* expression (Zhou et al., 1998). In our study, we demonstrated that homozygous alleles that contain strongly expressed reporter genes produce fewer RNAs than the hemizygous alleles. If the number of Pol II and PICs indeed works as the rate-limiting factor, all available proteins can bind to the promoter on the hemizygous, whereas they need to be divided between the two homologous alleles of homozygotes to initiate transcription, resulting in a lower transcription level.

Furthermore, the allelic competition observed in the “Enhancer Only” and “Promoter Only” constructs indicates that a common factor that binds to both the enhancer and the promoter may be responsible for the observed allelic interference (Figure 3B). Many papers have provided evidence that enhancers are actively transcribed through the binding of Pol II, Mediators, and other general TFs to the enhancer region, producing enhancer RNAs (eRNAs) (Kim et al., 2010, 2015; Arnold et al., 2020). Hence, we suggest that the limiting number of Pol II or PICs can lead to reduced transcriptional activity in homozygotes. In support of this hypothesis, the RNA production was comparable between the homozygous and hemizygous allele in NC13, when fewer genes are transcribed. In NC14, where thousands of genes are activated, the allelic interference was observed, and the degree of interference increased as the embryo progressed to late NC14 (Figure 3F). These results support our claim that the number of Pol II and PICs can become limiting in early embryos, affecting the allelic competition.

We acknowledge that thousands of genes are being transcribed in early embryos, and it is not intuitive to think that one additional transgene can affect the overall balance of TFs, Pol II, and PICs in each nucleus. Yet, others have reported similar phenomena of allelic competition, and we have also demonstrated that endogenous *snail* alleles interfere with each other (Figure 4) (Waymack et al., 2020, 2021). While strong physical interactions between an enhancer and the target promoter often result in high transcriptional activity (Symmons et al., 2016; Oudelaar et al., 2019), it was also demonstrated that the transcriptional efficiency is not linearly correlated with the degree of physical interactions (Zuin et al., 2022). We did not find direct evidence that the physical interaction between the two homologous alleles causes the reduced transcriptional activity in homozygous alleles. For example, transcriptional activity of the closely interacting homozygous alleles was similar to that of the homologous alleles located on the other side of the nucleus. Although the direct physical interactions in *trans* may not be affecting the reduced transcriptional activity in homologous alleles, we believe that different allelic displacement between the homozygous and hemizygous alleles could have affected their transcriptional efficiency.

We suggest that the localized clustering of Pol II and TFs in each nucleus allows allelic competition. Many recent papers have reported the presence of “transcription hubs” where TFs, Mediators, Pol II, and PICs form a cluster and the genes within each hub share the transcriptional machinery (Cisse et al., 2013; Mir et al., 2017; Cho et al., 2018; Yamada et al., 2019). According to the model, only a handful of each transcriptional machinery exists in a given hub, and adding one more reporter gene may work as a rate-limiting factor in this localized environment. The transcriptional machinery is non-uniformly distributed in a limited number of transcription hubs in a given nucleus (Edelman and Fraser, 2012; Mir et al., 2017; Boehning et al., 2018; Tsai et al., 2019; Yamada et al., 2019; Zhu et al., 2021). Even if numerous hubs exist in each nucleus, their positions could be relatively fixed and heterogeneously localized, preventing each hub to move freely toward active transcription loci. In our case, in the vicinity of the *MS2-yellow* reporter gene, there may exist just a single hub at that specific 3D location that is accessible by the reporter gene.

Taken together, we suggest that localized clusters of transcription hubs can limit the number of available molecules that bind to the enhancer and promoter regions, inducing allelic competition for strongly expressed genes. Our study provides additional insight into how the distribution of Pol II clusters in the nucleus and subsequent interallelic competitions can affect enhancer-mediated transcriptional regulation.

## Data Availability

The datasets presented in this study can be found in online repositories. The names of the repository/repositories and accession number(s) can be found below: https://github.com/limlab-upenn/deng2021.
