# Supplementary material for: Shared Transcriptional Machinery at Homologous Alleles Leads to Reduced Transcription in Early Drosophila Embryos
Source: Front Cell Dev Biol. 2022 Jul 11;10:912838. doi: 10.3389/fcell.2022.912838 (PMC9311490; doi:10.3389/fcell.2022.912838)
Supplement: Supplementary file 1 [file DataSheet1.PDF]

Supplementary Material

1 Supplementary Materials and Methods

1.1 Generation of transgenic and CRISPR fly lines

Pbphi vectors containing an attB site and the *MS2-yellow*-αtub 3′ UTR and *PP7-yellow*-αtub 3′ UTR reporter genes were used to generate transgenic lines used in this study (Fukaya et al., 2016). The 100bp core *sna* or *eve* promoter was amplified and inserted upstream of the *MS2* sequence. *sna* shadow enhancer was amplified using primers (5′ - GCA TTG AGG TGT TTT GTT - 3′) and (5′ - TAA ATT CCG ATT TTT CTT - 3′). Amplified enhancers were inserted either directly upstream of the *sna* promoter or downstream of the αtub 3′ UTR. “Enhancer Only” and “Promoter Only” constructs were generated by starting with the pbphi vector and inserting the *sna* shadow enhancer or the *eve* promoter-*PP7-yellow*-αtub 3′ UTR. The transgenic constructs were integrated into the fly genome using PhiC31-mediated site-specific integration, using the VK00033 and the VK00002 landing sites for the 3rd chromosome and 2nd chromosome insertion, respectively (Venken et al., 2006, 2009). The fly lines were generated by BestGene Inc.

Endogenous *sna-MS2* and *sna-PP7* fly lines were generated using CRISPR/Cas9-mediated homology-directed repair to insert 24 copies of MS2 and PP7 loops into the 3′UTR of the endogenous *sna* locus. The target site within the 3′UTR was selected using the flyCRISPR Target Finder (Gratz et al., 2014). The following gRNA oligo sequences were used: Sense (5′ - GTC GGG AAT AAT CTT AAC AAC AGT - 3′) and Antisense (5′ - AAA CAC TGT TGT TAA GAT TAT TCC - 3′).

1.2 Collection of homozygous and hemizygous embryos

Males containing the *PP7-yellow* reporter gene were crossed with female *nos>MCP-GFP*, *nos> mCherry-PCP*, *His2Av-eBFP2* flies (Lim et al., 2018). The progeny contains one copy of the *PP7-yellow* and one copy of *MCP-GFP*, *mCherry-PCP*, *His2Av-eBFP2*, and the females were crossed with the *MS2-yellow* containing males. 50% of the resulting embryos carry maternal *PP7-yellow* and paternal *MS2-yellow* reporter genes, which are referred to as “homozygotes.” The remaining 50% carry only the paternal *MS2-yellow* reporter gene, which are referred to as “hemizygotes.” All embryos have one maternal copy of the *MCP-GFP*, *mCherry-PCP*, and *His2Av-eBFP2*, which allows visualization of the *MS2-yellow* and *PP7-yellow* transcriptional activity under confocal microscope.

1.3 Live Imaging

Embryos were grown and collected at 23°C. Embryos were dechorionated using bleach, washed with water and mounted on a semipermeable membrane (Sarstedt AG & Co) for imaging. Live images were obtained from a Zeiss confocal microscope, LSM 800, with a Plan-Apochromat 40x1.3 NA oil objective. UV, 488 and 561 lasers were used to visualize His2AV-eBFP2, MCP-GFP and PCP-mCherry, respectively. The same laser settings were used for all images. The 16-bit images were taken with 1.1x zoom in a 145.20µm x 145.20µm x 9.75µm rectangular prism space where 14 Z-stack was obtained with 0.75µm steps. The temporal resolution was 27 seconds per frame.

1.4 Image analysis and statistical analysis

All the live images were processed and analyzed with Fiji and MATLAB (R2018b, MathWorks), using the custom-built code from previous studies (Keller et al., 2020). The p-values in each plot were calculated with the two-sided Wilcoxon rank sum test, which is equivalent to a Mann-Whitney U-test. The data and the Matlab codes used in the analysis are available in github (https://github.com/limlab-upenn/deng2021).

The raw images were maximum intensity Z-projected and concatenated into two-dimensional movies. Each nucleus was segmented by masks which were created in MATLAB and then manually corrected in Fiji. The fluorescent intensity within each nucleus mask at each time frame was extracted by taking the average of the two highest intensity via MATLAB. The histograms of the snapshots in Figure 1C and Movie 1 were adjusted for visualization purposes only. All analyses were performed on raw images. The error bars in Figure 3D were calculated from 1000 rounds of bootstrapping with subsample sizes of 50% of the active nuclei of each construct shown.

2 Supplementary Figures and Tables

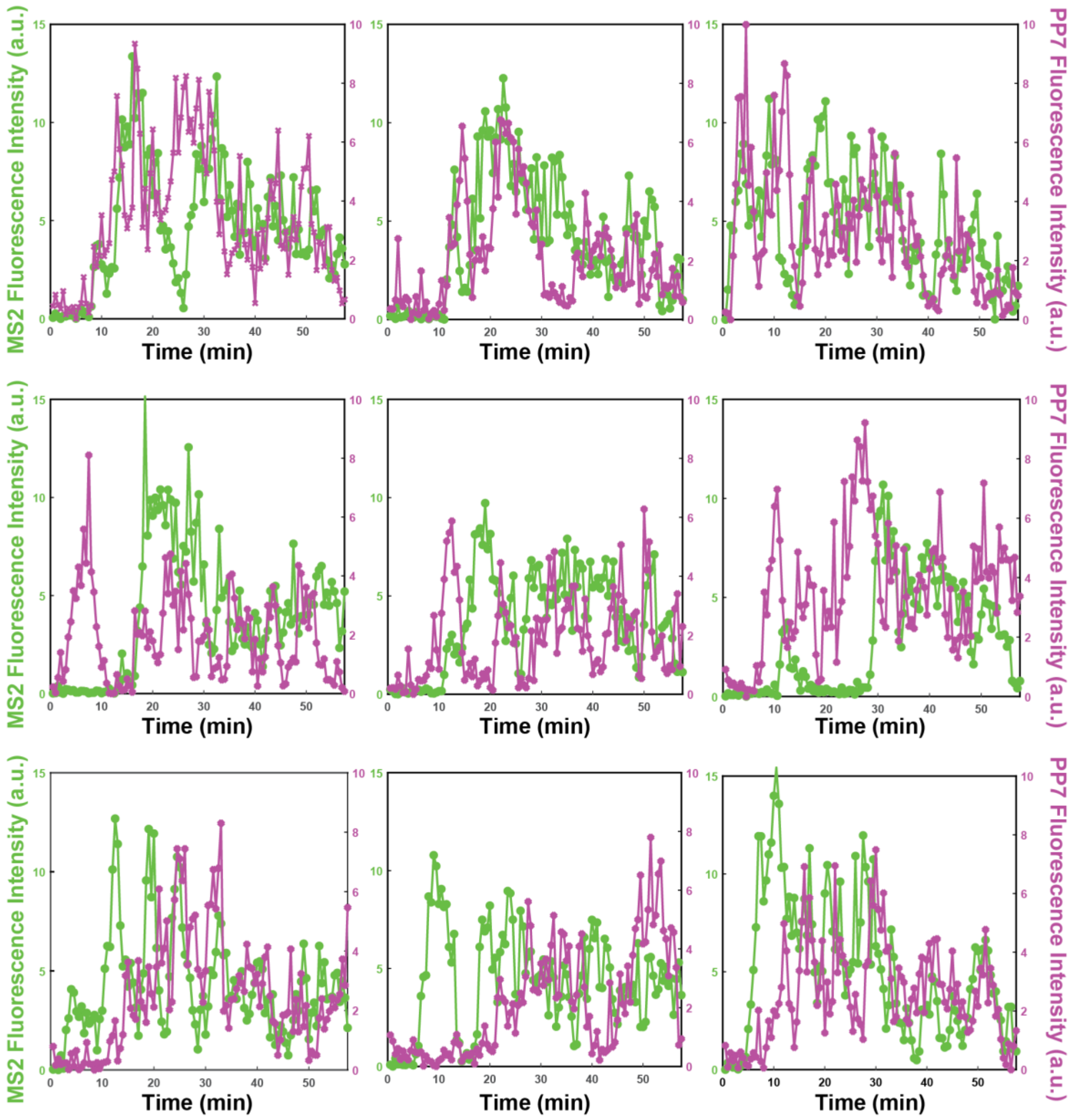

Figure S1. Representative transcriptional trajectories from one homozygous embryo.

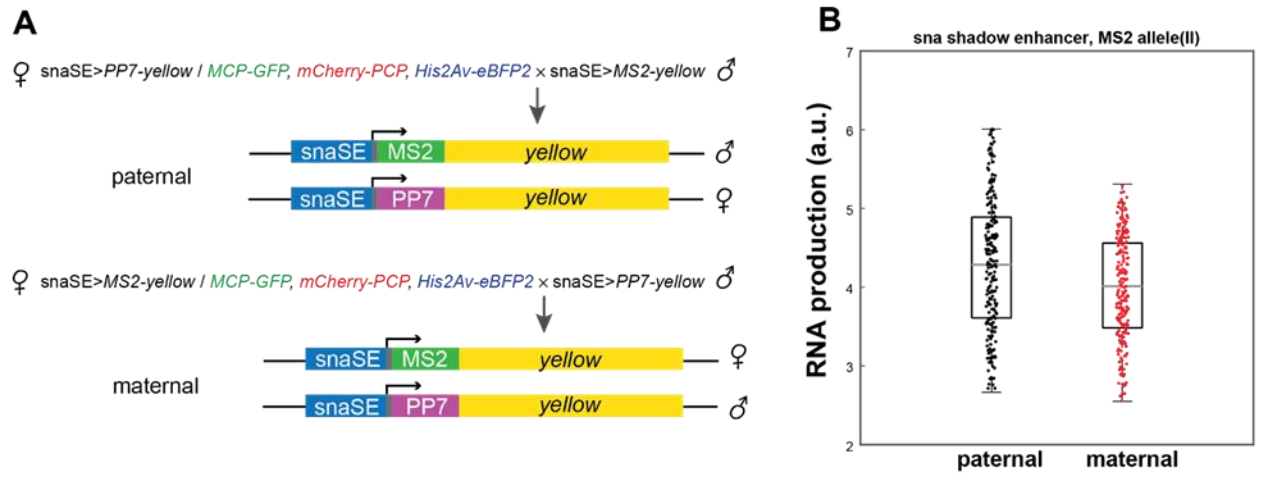

Figure S2. Comparison of the paternal and maternal snaSE>*MS2-yellow* allele. (A) Schematic of genetic crosses to generate embryos with the paternal (up) and the maternal (down) *MS2-yellow* allele. **(B)** RNA production from paternal and maternal snaSE>*MS2-yellow* allele in snaSE homozygous embryos.

Movie S1. Live imaging of hemizygous and homozygous snaSE>*yellow* embryos.

3 Supplementary References

Fukaya, T., Lim, B., and Levine, M. (2016). Enhancer Control of Transcriptional Bursting. *Cell* 166, 358–368. doi:10.1016/j.cell.2016.05.025.

Gratz, S. J., Ukken, F. P., Rubinstein, C. D., Thiede, G., Donohue, L. K., Cummings, A. M., et al. (2014). Highly Specific and Efficient CRISPR/Cas9-Catalyzed Homology-Directed Repair in Drosophila. *Genetics* 196, 961–971. doi:10.1534/genetics.113.160713.

Keller, S. H., Jena, S. G., Yamazaki, Y., and Lim, B. (2020). Regulation of spatiotemporal limits of developmental gene expression via enhancer grammar. *Proc. Natl. Acad. Sci. U. S. A.* 117, 15096–15103. doi:10.1073/PNAS.1917040117/SUPPL_FILE/PNAS.1917040117.SM04.MOV.

Lim, B., Heist, T., Levine, M., and Fukaya, T. (2018). Visualization of Transvection in Living Drosophila Embryos. *Mol. Cell* 70, 287-296.e6. doi:10.1016/j.molcel.2018.02.029.

Venken, K. J. T., Carlson, J. W., Schulze, K. L., Pan, H., He, Y., Spokony, R., et al. (2009). Versatile P[acman] BAC libraries for transgenesis studies in Drosophila melanogaster. *Nat. Methods* 6, 431–434. doi:10.1038/nmeth.1331.

Venken, K. J. T., He, Y., Hoskins, R. A., and Bellen, H. J. (2006). P[acman]: A BAC Transgenic Platform for Targeted Insertion of Large DNA Fragments in D. melanogaster. *Science (80-.).* 314, 1747–1751. doi:10.1126/science.1134426.
